# The ethics of ChatGPT in medicine and healthcare: a systematic review on Large Language Models (LLMs)

**DOI:** 10.1038/s41746-024-01157-x

**Published:** 2024-07-08

**Authors:** Joschka Haltaufderheide, Robert Ranisch

**Affiliations:** Faculty of Health Sciences Brandenburg, University of Potsdam, Am Mühlenberg 9, Potsdam, 14476 Germany

**Keywords:** Medical ethics, Ethics, Information technology

## Abstract

With the introduction of ChatGPT, Large Language Models (LLMs) have received enormous attention in healthcare. Despite potential benefits, researchers have underscored various ethical implications. While individual instances have garnered attention, a systematic and comprehensive overview of practical applications currently researched and ethical issues connected to them is lacking. Against this background, this work maps the ethical landscape surrounding the current deployment of LLMs in medicine and healthcare through a systematic review. Electronic databases and preprint servers were queried using a comprehensive search strategy which generated 796 records. Studies were screened and extracted following a modified rapid review approach. Methodological quality was assessed using a hybrid approach. For 53 records, a meta-aggregative synthesis was performed. Four general fields of applications emerged showcasing a dynamic exploration phase. Advantages of using LLMs are attributed to their capacity in data analysis, information provisioning, support in decision-making or mitigating information loss and enhancing information accessibility. However, our study also identifies recurrent ethical concerns connected to fairness, bias, non-maleficence, transparency, and privacy. A distinctive concern is the tendency to produce harmful or convincing but inaccurate content. Calls for ethical guidance and human oversight are recurrent. We suggest that the ethical guidance debate should be reframed to focus on defining what constitutes acceptable human oversight across the spectrum of applications. This involves considering the diversity of settings, varying potentials for harm, and different acceptable thresholds for performance and certainty in healthcare. Additionally, critical inquiry is needed to evaluate the necessity and justification of LLMs’ current experimental use.

## Introduction

Large language models (LLMs) have emerged as a transformative force in artificial intelligence (AI), generating significant interest across various sectors. The 2022 launch of OpenAI’s ChatGPT demonstrated their groundbreaking capabilities, revealing the current state of development to a wide audience. Since then, public availability and scientific interest have resulted in a flood of scientific papers exploring possible areas of application^1^ as well as their ethical and social implications from a practical perspective^2^. A particularly rapid adoption of LLMs is seen in medicine and healthcare^3^, encompassing clinical, educational and research applications^3–9^. This development may present a case where a general-purpose technology swiftly integrates into specific domains. According to Libsey, such technologies are characterized by their potential for extensive refinement and expansion, a wide array of applications across various processes, and significant synergies with existing technologies^10,11^. In a brief span, a significant number of publications have investigated the potential uses of LLMs in medicine and healthcare^12^, indicating a positive trajectory for the integration of medical AI. Present-day LLMs, such as ChatGPT, are considered to have a promising accuracy in clinical decision-making^13,14^, diagnosis^15^, symptom-assessment, and triage-advice^16^. In patient-communication, it has been posited that LLMs can also generate empathetic responses^17^. LLMs specifically trained on biomedical corpora forebode even further capacities for clinical application and patient care^18^ in the foreseeable future.

Conversely, the adoption of LLMs is entwined with ethical and social concerns^19^. In their seminal work, Bender et al. anticipated real-world harms that could arise from the deployment of LLMs^20^. Scholars have delineated potential risks across various application domains^21,22^. The healthcare and medical fields, being particularly sensitive and heavily regulated, is notably susceptible to ethical dilemmas. This sector is also underpinned by stringent ethical norms, professional commitments, and societal role recognition. Despite the potential benefits of employing advanced AI technology, researchers have underscored various ethical implications associated with using LLMs in healthcare and health-related research^4,6,7,23–26^. Paramount concerns include the propensity of LLMs to disseminate inadequate information, the input of sensitive health information or patient data, which raises significant privacy issues^24^, and the perpetuation of harmful gender, cultural or racial biases^27–30^, well known from machine learning algorithms^31^, especially in healthcare^32^. Case reports have documented that ChatGPT has already caused actual damage, potentially life-threatening for patients^33^.

While individual instances have drawn attention to ethical concerns surrounding the use of LLMs in healthcare, there appears to be a deficit in comprehensive, systematic overviews addressing these ethical considerations. This gap is significant, given the ambitions to rapidly integrate LLMs and foundational models into healthcare systems^34^. Our intention is to bridge this lacuna by mapping out the ethical landscape surrounding the deployment of LLMs in this field. To this end, we conducted a systematic review of the current literature including relevant databases and preprint servers. Our inquiry was structured around two research questions: Firstly, we sought to delineate the ethically relevant applications, interventions, and contexts where LLMs have been tested or proposed within the realms of medicine and healthcare. Secondly, we aimed to identify the principal outcomes as well as the opportunities, risks, benefits, and potential harms associated with the use of LLMs in these sectors, as deemed significant from an ethical standpoint. Through this, we aspire not only to outline the current ethical discourse but also to inform future dialogue and policy-making at the intersection of LLMs and healthcare ethics.

## Results

**Fig. 1:**
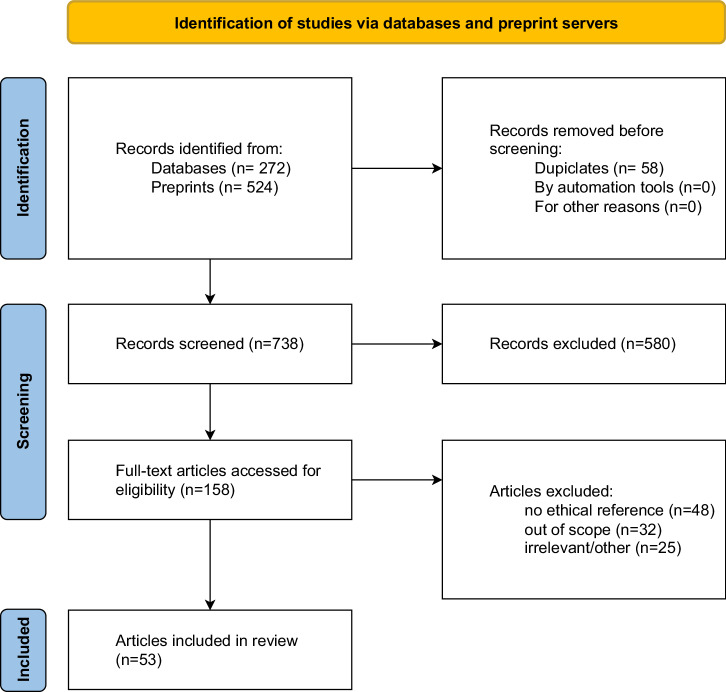
Flow of records through the screening process. This Diagram following PRISMA guidelines showing the flow of records through the screening process.

Our search yielded a total of 796 database hits. After removal of duplicates, 738 records went through title/abstract screening. 158 full-texts were assessed. 53 records were included in the dataset, encompassing 23 original articles^25,35–56^, including theoretical or empirical work, 11 letters^57–67^, six editorials^68–73^, four reviews^8^^,74–76^, three comments^24,77,78^, one report^79^ and five unspecified articles^80–84^. The flow of records through the review process can be seen in Fig. 1. Most works focus on applications utilizing ChatGPT across various healthcare fields, as indicated in Table 1. Regarding the affiliation of the first authors, 25 articles come from North America, 11 from Europe, six from West Asia, four from East asia, three from South Asia and four from Australia.

**Table 1:** Overview of the included records

Publication			Procedural Quality Control		Setting	
Title	Origin	Article Types	Peer Reviewed	COI	Device	Field of Application
Abdulai & Hung^77^	CAN	Commentary	Unclear	Unclear	ChatGPT; ChatGPT 4	Nursing education, research and practice
Agbavor & Liang^35^	USA	Empirical Article	Yes	None disclosed	GPT 3	Neurology
Ahn^57^	KOR	Letter	No	None disclosed	ChatGPT	Emergency Medicine
Ali et al.^36^	QAT	Theoretical Article	Preprint	Unclear	ChatGPT, Google Bard, Meta LLaMA	Healthcare
Almazyad et al.^37^	SAU	Empirical Article	Yes	Unclear	ChatGPT 4	Pediatric Palliative Care
Antaki et al.^38^	CAN	Empirical Article	Preprint	Unclear	ChatGPT; GPT 3.5	Ophtalmology
Arslan^58^	TUR	Letter	No	None disclosed	ChatGPT	Obesity Treatment
Beltrami & Grant-Kels^59^	USA	Letter	No	Conflict disclosed	ChatGPT	Dermatology
Buzzaccarini et al.^60^	ITA	Letter	No	Conflict disclosed	ChatGPT	Aesthetic Medicine
Carullo et al.^40^	ITA	Empirical Article	Yes	None disclosed	ChatGPT	Epidemiological Research
Cheng et al.^61^	CHN	Letter	No	None disclosed	ChatGPT; GPT 3	Infectiology
Connor & O’Neill^39^	IRL	Theoretical Article	Preprint	Unclear	ChatGPT; ChatDoctor; Google BARD	Sport Science and Medicine
Currie^80^	AUS	Unspecified	Yes	None disclosed	ChatGPT; GPT 3.5	Nuclear Medicine and Radiology
Dave et al.^8^	IND	Review	Yes	None disclosed	ChatGPT	Medicine
De Angelis et al.^46^	ITA	Theoretical Article	Yes	Conflict disclosed	GPT; BERT; GPT 2; GPT 3; GPT 4; Instruct GPT; BioBERT; BioGPT; PubMedGPT; Med-PaLm; CORD-19	Public Health
Eggman & Blatz^81^	USA	Unspecified	Unclear	None disclosed	ChatGPT	Dentistry
Ferrara^41^	USA	Theoretical Article	Preprint	Unclear	ChatGPT	Healthcare
Ferreira & Lippoff^78^	USA	Commentary	Unclear	None disclosed	ChatGPT	Dermatology
Gottlieb et al.^82^	USA	Unspecified	Yes	None disclosed	ChatGPT	Emergency Medicine
Guo et al.^42^	CAN	Empirical Article	Preprint	Conflict disclosed	ChatGPT; GPT 3; NeuroGPT-X	Neurosurgery
Guo et al.^79^	USA	Report	Preprint	Unclear	ProteinChat	Protein Research
Gupta et al.^62^	USA	Letter	No	None disclosed	ChatGPT	Aesthetic Surgery
Harrer^83^	AUS	Unspecified	Yes	Conflict disclosed	ChatGPT; LaMDA; BARD; Med-Palm	Healthcare
Harskamp & Clercq^43^	NDL	Empirical Article	Preprint	None disclosed	ChatGPT; InstructGPT	Cardiopulmonary Medicine
Hosseini et al.^44^	USA	Empirical Article	Preprint	Unclear	ChatGPT; GPT 4; Elicit; Med-PaLM	Education, Research and Healthcare
Howard et al.^63^	GBR	Letter	No	Conflict disclosed	ChatGPT	Infection Medicine
Jairoun et al.^68^	UAE	Editorial	No	Unclear	ChatGPT	Pharmacy
Kavian et al.^69^	USA	Editorial	No	None disclosed	ChatGPT	Surgery
Knebel et al.^45^	GER	Empirical Article	Preprint	None disclosed	ChatGPT; GPT 3	Ophtalmology
Li et al.^64^	CHN	Letter	No	No	ChatGPT	Surgery
Li et al.^24^	USA	Commentary	Unclear	No	ChatGPT; BioGPT; LaMDA; Sparrow; Pangu Alpha; OPT-IML; Megataron Turing MLG	Medicine and Medical Research
Padovan et al.^47^	ITA	Empirical Article	Preprint	None disclosed	ChatGPT	Occupational Medicine
Page et al.^70^	USA	Editorial	No	Conflict disclosed	ChatGPT 4	Microbial genomics research
Pal et al.^48^	IND	Empirical Article	Preprint	Unclear	BERT; BioBERT; BioClinicalBERT; SciBERT; UMLS-BERT	Medicine
Perlis^65^	USA	Letter	Preprint	Conflict disclosed	ChatGPT 4	Psychopharmacology
Rau et al.^49^	GER	Empirical Article	Preprint	Unclear	ChatGPT; GPT 3.5 Turbo; accGPT	Radiology
Sallam^74^	JOR	Review	Preprint	None disclosed	ChatGPT	Healthcare
Schmälzle & Wilcox^50^	USA	Theoretical Article	Yes	None disclosed	GPT 2	Public Health
Shariar & Hayawi^51^	CAN	Theoretical Article	Preprint	None disclosed	ChatGPT; BERT	Healthcare
Singh^71^	IND	Editorial	No	Unclear	ChatGPT	Mental Health
Snoswell et al.^84^	AUS	Unspecified	No	Unclear	ChatGPT	Pharmacy
Stewart et al.^52^	AUS	Theoretical Article	Preprint	None disclosed	BERT; various Natural language processing models	Healthcare
Suresh et al.^53^	USA	Empirical Article	Preprint	None disclosed	ChatGPT; GPT 4	Otolaryngology
Tang et al.^54^	USA	Empirical Article	Preprint	Unclear	ChatGPT; GPT 3.5	Medicine
Temsah et al.^75^	SAU	Review	Yes	None disclosed	ChatGPT	Healthcare and Health Research
Thomas^72^	USA	Editorial	No	Unclear	ChatGPT	Mental Health Nursing
Waisberg et al.^66^	IRL	Letter	No	None disclosed	ChatGPT; GPT 4	Opthalmology
Xie & Wang^76^	USA	Review	Preprint	None disclosed	BERT; BioBERT; BlueBERT; PubMedBERT; ChatGPT; GPT 4; BioGPT; Med-PaLM	Healthcare and Medicine
Yeo et al^56^	USA	Empirical Article	Preprint	None disclosed	ChatGPT; GPT 3.5	Hepatology
Yeo et al.^55^	USA	Empirical Article	Preprint	None disclosed	ChatGPT; GPT 4	Hepatology
Yeung et al.^28^	GBR	Empirical Article	Preprint	Unclear	ChatGPT; Foresight; PaLM, Gopher; Chinchilla	Medicine
Yoder-Wise^73^	USA	Editorial	No	None disclosed	ChatGPT	Nursing
Zhong et al.^67^	CHN	Letter	No	None disclosed	ChatGPT	Neuropsychiatric practice and research

During analysis, four general themes emerged in our dataset, which we used to structure our reporting. These themes include clinical applications, patient support applications, support of health professionals, and public health perspectives. Table 2 provides exemplary scenarios for each theme derived from the dataset.

**Table 2:** Exemplary applications of LLMs

Predictive Analysis and Risk Assessment	
Connor & O’Neill^39^	Supporting initial diagnosis and triaging of patients by fine-tuning LLMs on a specialised dataset of electronic medical records, clinical notes, sports science and medicine literature.
Stewart et al.^52^	Using traditional and modern natural language processing to triage patients on arrival based on structured data and unstructured free-text history of presenting complaint to predict risk stratification. This includes predictions on the likelihood of admission to hospital, prediction of critical illness, prediction of triage score, prediction of provider-assigned chief complaint, prediction of investigation, and prediction of infection.
Patient Consultation and Communication	
Buzzaccarini et al.^60^	Enhancing patient consultations by providing accurate and reliable information on aesthetic procedures, their risks, benefits and potential outcomes, enabling well-informed decisions and improved treatment outcomes.
Currie^80^	Providing language translation and helping health professionals to communicate with patients speaking foreign languages; helping health professionals to educate their patients and empower patients to take an active role.
Public Health	
Schmälzle & Wilcox^50^	Using LLMs to create an AI-guided message creation system to disseminate health-related information via social media.
Cheng et al.^61^	Using ChatGPT to monitor news and social media platforms for signs of outbreaks of disease clusters and to alert health professionals to potential threats.
Diagnosis	
Agbavor & Liang^35^	Using GPT 3 to distinguish individuals with Alzheimer's disease from healthy controls and to infer cognitive testing scores based on linguistic features. It is shown that this approach outperforms conventional approaches and performs comparable to specifically fine-tuned models. Usable as a web app in a doctor's office.
Rau et al.^49^	Supporting radiologists' diagnostic performance by providing imaging recommendations in accordance with recent guidelines.
Treatment Planning	
Arslan^58^	Using ChatGPT to provide personalized recommendations on topics such as nutrition, exercise and psychological support in obesity treatment.
Cheng et al.^61^	Using ChatGPT to provide treatment recommendations based on patients clinical presentations, disease severity, and comorbidities.
Patient Support	
Yeo et al.^55^	Using ChatGPT as an informational plattform to comprehend and to respond to cirrhosis related questions in different languages, adressing barriers that may impact patient care.
Knebel et al.^45^	Using ChatGPT for the assessment of acute ophtalmological conditions with regard to triage accurracy and recommendations for preclinical measures.
Professional Support and Research	
Hosseini et al.^44^	Using LLMs to increase efficiency in note-taking through prepopulation of forms, voice recording and converting recordings into clinical notes, or synthesizing existing patient notes to save clinicians' time.
Gottlieb et al.^82^	Using Conversational AI to create study documents by translating complex concepts into simpler ones or designing informed consent documents for patients.
Guo et al.^79^	Using a ChatGPT-like (ProteinGPT) systems to accelerate protein research. The model is aimed at learning and understanding protein 3D structures. ProteinGPT enables users to upload proteins, ask questions, and engage in interactive conversations to gain insights.

### Clinical applications

To support initial diagnosis and triaging of patients^39,52^, several authors discuss the use of LLMs in the context of predictive patient analysis and risk assessment in or prior to clinical situations as a potentially transformative application^74,80^. The role of LLMs in this scenario is described as that of a “co-pilot” using available patient information to flag areas of concern or to predict diseases and risk factors^44^.

Currie, in line with most authors, notes that predicting health outcomes and relevant patterns is very likely to improve patient outcomes and contribute to patient benefit^80^. For example, overcrowded emergency departments present a serious issue worldwide and have a significant impact on patient outcomes. From a perspective of harm avoidance, using LLMs with triage notes could lead to reduced length of stay and a more efficient utilization of time in the waiting room^52^.

All authors note, however, that such applications might also be problematic and require close human oversight^39,44,51,80^. Although LLMs might be able to reveal connections between disparate knowledge^40^, generating inaccurate information would have severe negative consequences^44,74^. This could lead to direct harm to patients or provide clinicians with false and dangerous justifications and rationales for their decisions^74^. These problems are tightly connected to inherent biases in LLMs, their tendency to “hallucinate” and their intransparency^52^. The term “hallucination” refers to an LLM generating plausible and often confident statements that are factually incorrect in the sense of not being grounded in the data^85^. In addition, uncertainties are increased by use of unstructured data. Medical notes often differ from the data pretrained models utilise. This makes it difficult to predict accuracy of output when using such data in prompts or for fine-tuning LLMs^52^. The interpretability of results and recommendations introduce additional complexity and sources of potential harm^52^. Currie notes that despite such difficulties, the use of LLMs proceeds largely in absence of guidelines, recommendations and control. The outcome, hence, ultimately depends on clinicians’ ability to interpret findings and identify inaccurate information^80^.

In patient consultation and communication, LLMs can offer a novel approach to patient-provider interaction by facilitating informational exchange and bridging gaps between clinical and preclinical settings, such as self-management measures or community aids^8^. This includes easing the transition between settings by removing barriers to communication^44,60,80,83^ or removing barriers in the clinical workflow to facilitate timely and efficient support. As is suggested, LLMs can collect information from patients or provide additional information, enabling well-informed decisions and increasing satisfaction in patients^56,60,80^. Provision of language translation and simplification of medical jargon may allow patients to become more engaged in the process and enhance patient-provider communication^80,83^. However, it remains unclear in our dataset what such applications would look like in practice — specifically where, when and how LLMs could actually be integrated.

These suggestions necessitate consideration of ethically relevant boundaries regarding the protection of patient data, and safety^36,60,77,83^, potentially unjust disparities^36,60,83^, and the broader dimensions of care, such as the therapeutic relationship^36,59,61,64,77^. Robust measures to avoid incorrect information in technological mediation of communication and the need to strike a balance with “the human touch” of care^60^ are stressed. With regard to the former, Buzzaccarini et al. argue for robust expert oversight. Regarding the latter, Li et al. note a potential shift in power dynamics between patients and providers, in which providers might lose their authoritative position and might be seen as less knowledgeable^64^. Others fear a loss of personal care that should be avoided^36,61,77^ and the lack of contextual content of individual health challenges^42,77^. Open communication and consent to the technical mediation of patient-provider communication are required to promote trust but might be difficult to achieve^69,78^.

Many studies in our dataset discuss the possible use of LLMs for diagnosis^8,36,39,44,59,61,66,67,74,75,78,80^. It is suggested that the LLMs’ ability to analyze large amounts of unstructured data provides pathways to timely, efficient and more accurate diagnosis to the benefit of patients^35,36,67,75,78^. It might also enable the discovery of hidden patterns^39^ and reduce healthcare costs^36,49^.

An ethical problem emerges with potentially negative effects on patient outcomes due to biases in the training data^36,39,41,74,75,78^, especially with the lack of diverse datasets risking the underrepresentation of marginalized or vulnerable groups. Biased models may result in unfair treatment of disadvantaged groups, leading to disparities in access, exacerbating existing inequalities, or harming persons through selective accuracy^41^. Based on an experimental study setup, Yeung et al. deliver an insightful example showing that ChatGPT and Foresight NLP exhibit racial bias towards Black patients^28^. Issues of interpretability, hallucinations, and falsehood mimicry exacerbate these risks^35,36,44,74^. With regard to transparency, two sources suggest that LLM-supported diagnoses hamper the process of providing adequate justification due to their opacity^36,74^. This is understood to threaten the authoritative position of professionals, leaving them at risk of not being able to provide a rationale for a diagnosis^35^ and might lead to an erosion of trust between both parties. This is in line with others noting that LLMs are not able to replicate a process of clinical reasoning in general and, hence, fail to comprehend the complexity of the process^44,59,75^. Based on the principle of avoidance of harm, it is an important requirement to subject each generated datum to clinical validation as well as to develop “ethical and legal systems” to mitigate these problems^36,39,59^.

It needs to be noted, however, that the technically unaided process of diagnosis is also known to be subjective and prone to error^67^. This implies that an ethical evaluation should be carried out in terms of relative reliability and effectiveness compared to existing alternatives. Whether and under what circumstances this might be the case is a question that is not addressed.

Six studies in our dataset highlight the use of LLMs in providing personalized recommendations for treatment regimens or to support clinicians in treatment decisions based on electronic patient information or history^58,60,61,66,67,80^, providing a quick and reliable course of action to clinicians and patients. However, as with diagnostic applications, biases and perpetuating existing stereotypes and disparities are a constantly discussed theme^60,61,67^. Ferrara also cautions that LLMs will likely prioritize certain types of treatments or interventions over others, disproportionately benefiting certain groups and disadvantaging others^41^.

Additionally, it is highlighted that processing patient data raises ethical questions regarding confidentiality, privacy, and data security^58,60,61,66,67^. This especially applies to commercial and publicly available models such as ChatGPT. Inaccuracies in potential treatment recommendations are also noted as a concerning source of harm^58,60,61,66,67^. In a broader context, several authors suggest that for some LLMs, the absence of internet access, insufficient domain-specific data, limited access to treatment guidelines, lack of knowledge about local or regional characteristics of the healthcare system, and outdated research significantly heighten the risk of inaccurate recommendations^24,37,38,40,47,55^.

### Patient support applications

Almost all authors concerned with patient-facing applications highlight the benefits of rapid and timely information access that users experience with state-of-the-art LLMs. Kavian et al. compare patients’ use of chatbots with shifts that have accompanied the development of the internet as a patient information source^69^. Such access can improve laypersons’ health literacy by providing a needs-oriented access to comprehensible medical information^68^, which is regarded as an important precondition of autonomy to allow more independent, health-related decisions^8,74^. In their work on the use of ChatGPT 4 in overcoming language barriers, Yeo et al. highlight an additional benefit, as LLMs could provide cross-lingual translation and thus contribute to equalizing healthcare and racial disparities^56^.

Regarding ethical concerns and risks, biases are seen as a significant source of harm^8,39,74,75^. The literature also highlights a crucial difference in the ethical acceptability of using patient support applications, leading to a more critical stance when LLMs are used by laypersons compared to health professionals^28,53^. However, ethical acceptability varies across fields; for instance, otolaryngology and infectious disease studies find ChatGPT’s responses to patients lack detail but aren’t harmful^53^, whereas pharmacology and mental health indicate greater potential risks^67,68^.

LLMs can offer laypersons personalized guidance, such as lifestyle adjustments during illness^80^, self-assessment of symptoms^61,63^, self-triaging, and emergency management steps^8,57^. Although current arrangements seem to perform well and generate compelling responses^8,47,63^, a general lack of situational awareness is noted as a common problem that might lead to severe harm^8,61,63^. Situational awareness means the ability to generate responses based on contextual criteria such as the personal situation, medical history or social situation. The inability of most current LLMs to seek clarifications by asking questions and their lack of sensitivity to query variations can lead to imprecise answers^45,63^. For instance, research by Knebel et al. on self-triaging in ophthalmologic emergencies indicates that ChatGPT’s responses can’t reliably prioritize urgency, reducing their usefulness^45^.

### Support of health professionals and researchers

LLMs could automate administrative or documentation tasks like medical reporting^80^, or summarizing patient interactions^8^ including automatic population of forms or discharge summaries. The consensus is that LLMs could streamline clinical workflows^8,36,43,51,52,60,68,74,80,81,83^, offering time savings for health professionals currently burdened with extensive administrative duties^68,83^. By automating these repetitive tasks, professionals could dedicate more time to high-quality medical tasks^83^. Crucially, such applications would require the large-scale integration of LLMs into existing clinical data systems^49^.

In health research, LLMs are suggested to support text, evidence or data summarization^54,64,82^, identify research targets^8,61,72,83^, designing experiments or studies^72,83^, facilitate knowledge sharing between collaborators^37,70,80^, and to communicate results^74^. This highlights the potential for accelerating research^46,79^ and relieving researchers of workload^8,40,64,74,75,83^, leading to more efficient research workflows and allowing researchers to spend less time on burdensome routine work^8,80^. According to certain authors, this could involve condensing crucial aspects of their work, like crafting digestible research documents for ethics reviews or consent forms^82^. However, LLMs capacities are also critically examined, with Tang et al. emphasizing ChatGPT’s tendency to produce attribution and misinterpretation errors, potentially distorting original source information. This echoes concerns over interpretability, reproducibility, uncertainty handling, and transparency^54,74^.

Some authors fear that using LLMs could compromise research integrity by disrupting traditional trust factors like source traceability, factual consistency, and process transparency^24^. Additionally, concerns about overreliance and deskilling are raised, as LLMs might diminish researchers’ skills and overly shape research outcomes^46^. Given that using such technologies inevitably introduces biases and distortions to the research flow, Page et al. suggest researchers must maintain vigilance to prevent undue influence from biases introduced by these technologies, advocating for strict human oversight and revalidation of outputs^70^.

### Public health perspectives

The dataset encompasses studies that explore the systemic implications of LLMs, especially from a public health perspective^50,61,75^. This includes using LLMs in public health campaigns, for monitoring news and social media for signs of disease outbreaks^61^ and targeted communication strategies^50^. Additionally, research examines the potential for improving health literacy or access to health information, especially in low-resource settings. Access to health information through LLMs can be maintained free of charge or at very low costs for laypersons^55^. Considering the case of mental health, especially low- and middle-income countries might benefit^71^. These countries often have a huge treatment gap driven by a deficit in professionals or inequitable resource distribution. Using LLMs could mitigate accessibility and affordability issues, potentially offering a more favorable alternative to the current lack of access^71^.

However, a number of authors raise doubts about overly positive expectations. Schmälzle & Wilcox highlight the risks of a dual use of LLMs^50^. While they might further equal access to information, malicious actors can and seem to be using LLMs to spread fake information and devise health messages at an unprecedented scale that is harmful to societies^50,51,75^. De Angelis et al. take this concern one step further, presenting the concept of an AI-driven infodemic^46^ in which the overwhelming spread of imprecise, unclear, or false information leads to disorientation and potentially harmful behavior among recipients. Health authorities have often seen AI technologies as solutions to information overload. However, the authors caution that an AI-driven infodemics could exacerbate future health threats. While infodemic issues in social media and grey literature are noted, AI-driven infodemics could also inundate scientific journals with low-quality, excessively produced content^46^.

The commercial nature of most current LLM systems present another critical consideration. The profit-driven nature of the field can lead to concentrations of power among a limited number of companies and a lack of transparency. This economic model, as highlighted by several studies, can have negative downstream effects on accessibility and affordability^24,36,43^. Developing, using, or refining models can be expensive, limiting accessibility and customization for marginalized communities. Power concentration also means pricing control lies with LLM companies, with revenues predominantly directed towards them^44^. These questions are also mirrored in the selection of training data and knowledge bases^24^ which typically encompass knowledge from well-funded, English speaking countries and, thus, significantly underrepresents knowledge from other regions. This could exacerbate health disparities by reinforcing biases rather than alleviating them.

## Discussion

**Fig. 2:**
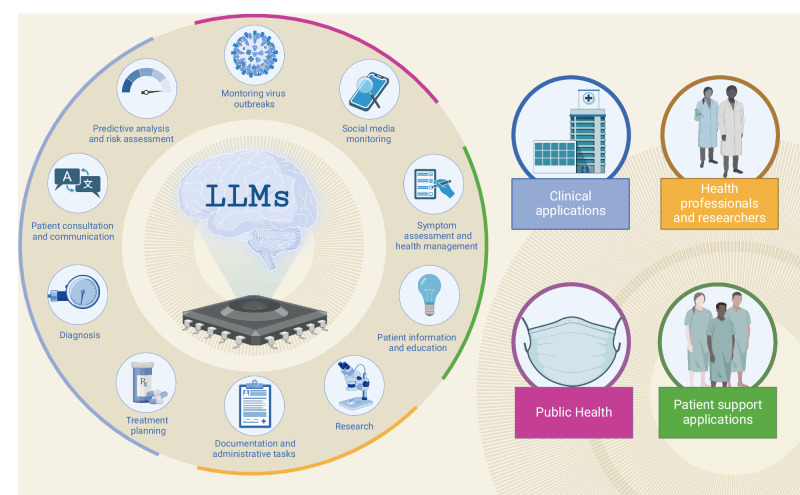
Fields of application of LLMs in medicine and healthcare. This figure shows the categories and subcategories of applications of LLMs.

Our analysis has unveiled an extensive range of LLM applications currently under investigation in medicine and healthcare (see Fig. 2). This surge in LLMs was largely caused by the advent and ease of use of ChatGPT, a platform not originally tailored for professional healthcare settings, yet widely adopted within it^12,83^. This presents a rather unique instance where a general-purpose technology has rapidly permeated the sector of healthcare and research to an unprecedented extent.

Our review highlights a vivid testing phase of LLMs across various healthcare domains^12^. Despite the lack of real-world applications, especially in the clinic, there is an overarching sentiment of the promise LLMs hold. It is posited that these tools could increase the efficiency of healthcare delivery and research, with the potential to benefit patient outcomes while alleviating the burdensome workload of healthcare professionals. These advantages of LLMs are largely attributed to their capabilities in data analysis, personalized information provisioning, and support in decision-making, particularly where quick analysis of voluminous unstructured data is paramount. Moreover, by mitigating information loss and enhancing medical information accessibility, LLMs stand to significantly bolster healthcare quality.

**Fig. 3:**
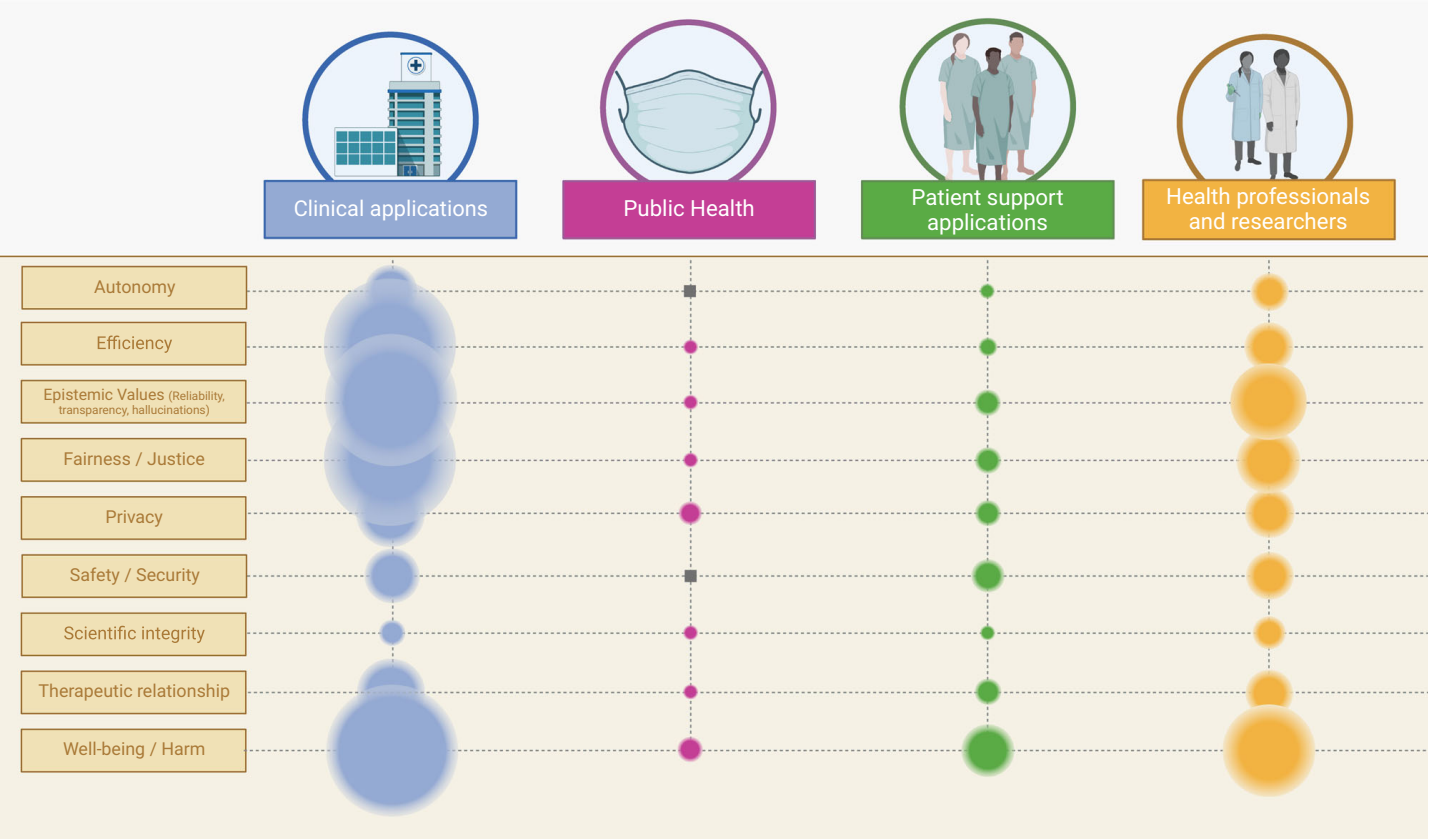
Discussed dimensions of impact of LLMs. This figure shows recurring ethical issues and their relative weight in each field of application based on the number of codes extracted during the analysis.

However, our study has also surfaced recurrent ethical concerns associated with LLMs. These concerns echo the wider discourse on AI ethics^86–88^, particularly in healthcare^89^, and touch on issues of fairness, bias, non-maleficence, transparency, and privacy. Yet, LLMs introduce a distinctive concern linked to a dimension of epistemic values, that is, their tendency to produce harmful misinformation or convincingly but inaccurate content through hallucinations as illustrated in Fig. 3^90^. The effects of such misinformation are particularly severe in healthcare, where the outcome could be dire. The inherent statistical and predictive architecture combined with the intransparency of LLMs presents significant hurdles in validating the clinical accuracy and reliability of their outputs^91–93^.

The inclination of LLMs to output erroneous information underscores the need for human oversight and continual validation of machine-generated output, as our dataset demonstrates. This need is accentuated by the lack of professional guidelines or regulatory oversight within this field^23^. Consequently, there is a noticeable demand for ethical guidelines, as evidenced within the literature surrounding healthcare applications of LLMs^46,60,64,70,71,74,75,78^.

### Future directions of ethics research

While we concur with the need for such guidance, our analysis suggests that the real challenge lies not in the articulation of such a need but in comprehending the scope of what this entails. There are inherent and contextual limitations and benefits associated with LLMs that warrant consideration. Inherently, state-of-the-art LLMs carry the risks of biases, hallucinations, and challenges in validity assessment, reliability testing, and reproducibility. Contextually, the effectiveness of LLM usage hinges on various situational factors, including the user utilizing LLMs, their level of expertise as well as their epistemic position (e.g. expert versus layperson), the specific domain of application, the risk profile of the application, and potential alternatives that the LLM is compared against.

A nuanced ethical discourse must recognize the multilayered nature of LLM–usage, from the epistemic stance of the user to the potential for harm, the varying degrees of potential harm due to misinformation or bias, and the diverse normative benchmarks for performance and acceptable levels of uncertainty. Our recommendation is to reframe the ethical guidance debate to focus on defining what constitutes acceptable human oversight and validation across the spectrum of applications and users. This involves considering the diversity of epistemic positions of users, the varying potentials for harm, and the different acceptable thresholds for performance and certainty in diverse healthcare settings. Such an approach should align with context-sensitive and participatory strategies for advancing technological development.

Given these questions, a critical inquiry is necessary into the extent to which the current experimental use of LLMs is both necessary and justified. Our dataset exemplifies a diversity of perspectives, methodologies, and applications of LLMs, revealing a significant degree of ambiguity and uncertainty about the appropriate engagement with this technology. Notably, a portion of current research seems propelled more by a sense of experimental curiosity than by well-defined methodological rigor, at times pushing the boundaries of ethical acceptability, particularly when sensitive real patient data are utilized to explore capabilities of systems like ChatGPT.

To frame these developments, it is instructive to consider the implementation of LLMs as a form of “social experiment”^94,95^. We employ this concept in a descriptive sense to denote a situation in which – according to van der Poel – the full benefits, risks, and ethical issues of a technology become evident only after its widespread adoption^95^. This perspective acknowledges the inherent uncertainties associated with the deployment of LLMs in medicine and healthcare due to their novelty, complexity, and opacity. Consequently, it necessitates that these technologies be introduced through an iterative process, which constitutes a learning endeavor. This approach facilitates a gradual understanding of the actual consequences of LLM use, thereby mitigating uncertainties. Furthermore, framing the current developments as social experiment also reinforces the need to establish and respect ethical limits – especially within the healthcare domain, where professional duties and responsibilities towards patients are foundational.

With this in mind, we suggest that understanding how we acquaint ourselves with disruptive technologies must be central to any future ethical discourse. There is a compelling need for additional research to ascertain the conditions under which LLMs can be appropriately utilized in healthcare, but also to establish conditions of gradual experimentation and learning that align with principles of health ethics.

### Limitations

This review addresses ethical considerations of using LLMs in healthcare at the current developmental stage. However, several limitations are important to acknowledge. Ethical examination of LLMs in healthcare is still nascent and struggles to keep pace with rapid technical advancements. Thus, the review offers a starting point for further discussions. A significant portion of the source material originated from preprint servers and did not undergo rigorous peer review, which can lead to limitations in quality and generalisability. Additionally, the findings’ generalizability may be limited due to variations in researched settings, applications, and interpretations of LLMs. Finally, we note a potential underrepresentation in our dataset, when it comes to non-Western perspectives. Most articles are affiliated with North American or European institutions. This might have an impact on the scope of ethical issues discussed as well as on how certain issues are addressed and evaluated. For example, many authors express hopes that LLMs might help to mitigate issues of global health justice such as unequal distribution of access to healthcare or treatment gaps in disadvantaged countries. However, a lack of more critical perspectives potentially informed by non-Western experience and exploration of LLMs needs to be noted. This includes, for example, addressing the implications of Western economic dominance or the effects of training data that predominantly represents Western populations. With this in mind, we do not understand our overview of ethical issues as exhaustive.

## Methods

### Protocol and registration

A review protocol focusing on practical applications and ethical considerations grounded in experience was designed by the authors and registered in the international prospective register of systematic reviews^96^. Ethical approval or consent to participate was not required for this systematic review.

### Information sources and search strategy

Relevant publication databases and preprint servers were queried (see Table 3 for sources).

**Table 3:** Overview on sources and search string

Sources	
Databases	MEDLINE via PubMed
	CINAHL
	Embase
	Philosophers’ Index
	PsychInfo
	IEEEX Xplore

Search	
Searchstring^1^	1. ChatGPT [Text Word]
	2. LLM [Text Word]
	3. Large Language Model [Text Word]
	4. 1 OR 2 OR 3
	4. Ethics [Text Word]
	5. Moral [Text Word]
	6. 4 OR 5
	7. 3 AND 6

^1^Wildcards and database-specific truncations (e.g. ethic*, moral*) where used where appropriate and applicable.

The decision to include preprint servers as well as databases was made based on the hypothesis that preprints are very common in technology-oriented fields. In addition, we hypothesized that even a mild publication delay would have prevented relevant work from already being indexed in the databases at the time of our search.

### Study selection

Inclusions were screened and extracted in a two-staged process following a modified rapid review approach^97^. Inclusion and exclusion criteria were based on the three key concepts of intervention, application setting, and outcomes (see Supplementary Note 1). No additional inclusion or exclusion criteria (e.g. publication type) were applied. However, we excluded work that was solely concerned with (ethical) questions of medical education, academic writing, authorship and plagiarism. While we recognize that these issues are affected by the use of LLMs in significant ways^6,98,99^ these, challenges are not specific to health-related applications.

### Data Collection and Extraction

Database searches were conducted in July 2023. Subsequently, the authors independently screened titles and abstracts of 10% of all database hits (73 records) to test and refine inclusion and exclusion criteria. After a joint discussion of the results, the remaining 90% were screened by the first author. Data was extracted using a self-designed extraction form (see Supplementary Note 2). The extraction categories were transformed into a coding tree using MaxQDA. Both authors independently coded 10% of the material to develop and refine the coding scheme in more detail. The remaining material was extracted by J.H. Results were iteratively discussed in three joint coding sessions.

### Synthesis

A final synthesis was conducted following a meta-aggregative approach. Based on our extraction fields, we, first, developed preliminary categories encompassing actors, values, device properties, arguments, recommendations and conclusions. These categories were, then, iteratively refined and aggregated through additional coding until saturation was reached.

### Quality appraisal

Given the constraints of normative quality appraisal^100^ and in line with our research goal to portrait the landscape of ethical discussions, we decided to take a hybrid approach to the quality question. We descriptively report on procedural quality criteria (see Table 1) to distinguish material that underwent processual quality control (such as peer review) from other material. In addition, we critically engage with the findings during reporting to appraise comprehensiveness and validity of the extracted information pieces.

### Reporting summary

Further information on research design is available in the Nature Research Reporting Summary linked to this article.

### Supplementary information

Supplementary Information

Reporting Summary

Confirmation of Publication and Licensing Rights

Confirmation of Publication and Licensing Rights

## Data Availability

The datasets used and/or analysed during the current study are available from the corresponding author on reasonable request.
