# Supplementary material for: The ethics of ChatGPT in medicine and healthcare: a systematic review on Large Language Models (LLMs)
Source: NPJ Digit Med. 2024 Jul 8;7:183. doi: 10.1038/s41746-024-01157-x (PMC11231310; doi:10.1038/s41746-024-01157-x)
Supplement: Supplementary file 2 — Reporting Summary [file 41746_2024_1157_MOESM2_ESM.pdf]

nature portfolio

Corresponding author(s): Robert Ranisch

Last updated by author(s): 14.05.2024

Reporting Summary

Nature Portfolio wishes to improve the reproducibility of the work that we publish. This form provides structure for consistency and transparency in reporting. For further information on Nature Portfolio policies, see our Editorial Policies and the Editorial Policy Checklist.

Statistics

For all statistical analyses, confirm that the following items are present in the figure legend, table legend, main text, or Methods section.

n/a	Confirmed	
☒	☐	The exact sample size (*n*) for each experimental group/condition, given as a discrete number and unit of measurement
☒	☐	A statement on whether measurements were taken from distinct samples or whether the same sample was measured repeatedly
☒	☐	The statistical test(s) used AND whether they are one- or two-sided<br>*Only common tests should be described solely by name; describe more complex techniques in the Methods section.*
☒	☐	A description of all covariates tested
☒	☐	A description of any assumptions or corrections, such as tests of normality and adjustment for multiple comparisons
☒	☐	A full description of the statistical parameters including central tendency (e.g. means) or other basic estimates (e.g. regression coefficient) AND variation (e.g. standard deviation) or associated estimates of uncertainty (e.g. confidence intervals)
☒	☐	For null hypothesis testing, the test statistic (e.g. *F*, *t*, *r*) with confidence intervals, effect sizes, degrees of freedom and *P* value noted<br>*Give P values as exact values whenever suitable.*
☒	☐	For Bayesian analysis, information on the choice of priors and Markov chain Monte Carlo settings
☒	☐	For hierarchical and complex designs, identification of the appropriate level for tests and full reporting of outcomes
☒	☐	Estimates of effect sizes (e.g. Cohen's *d*, Pearson's *r*), indicating how they were calculated

Our web collection on statistics for biologists contains articles on many of the points above.

Software and code

Policy information about availability of computer code

Data collection	During Abstract and title screening colandr was used (https://colandrapp.com).
Data analysis	Extraction and syntesis was conducted using MaxQDA 20.

For manuscripts utilizing custom algorithms or software that are central to the research but not yet described in published literature, software must be made available to editors and reviewers. We strongly encourage code deposition in a community repository (e.g. GitHub). See the Nature Portfolio guidelines for submitting code & software for further information.

Data

Policy information about availability of data

All manuscripts must include a data availability statement. This statement should provide the following information, where applicable:

- Accession codes, unique identifiers, or web links for publicly available datasets
- A description of any restrictions on data availability
- For clinical datasets or third party data, please ensure that the statement adheres to our policy

The datasets used and/or analysed during the current study are available from the corresponding author on reasonable request.

Research involving human participants, their data, or biological material

Policy information about studies with human participants or human data. See also policy information about sex, gender (identity/presentation), and sexual orientation and race, ethnicity and racism.

Reporting on sex and gender	This study is based on a secondary data analysis using qualitative methods. Categories of sex and gender were considered insofar as they emerged in connection with ethical issues in the primary material. No statistical analysis with regard to these categories was performed due to the qualitative method of analysis.
Reporting on race, ethnicity, or other socially relevant groupings	This study is based on a secondary data analysis using qualitative methods. Categories of race and ethnicity were considered insofar as they emerged in connection with ethical issues (esp. marginalization of groups and biases in training data) in the primary material. Categories of race and ethnicity are used in reference to their meaning in the primary material.
Population characteristics	No human research participants were involved in this study.
Recruitment	No human research participants were involved in this study.
Ethics oversight	Ethical approval or consent to participate was not required for this systematic review.

Note that full information on the approval of the study protocol must also be provided in the manuscript.

Field-specific reporting

Please select the one below that is the best fit for your research. If you are not sure, read the appropriate sections before making your selection.

☐ Life sciences ☒ Behavioural & social sciences ☐ Ecological, evolutionary & environmental sciences

For a reference copy of the document with all sections, see nature.com/documents/nr-reporting-summary-flat.pdf

Behavioural & social sciences study design

All studies must disclose on these points even when the disclosure is negative.

Study description	A systematic review was conducted gathering information about current state of deployment of LLMs in healthcare and ethical issues discussed or uncovered in literature. Studies from databases and preprint servers were screened and extracted following a modified rapid review approach. Methodological quality was assessed using a hybrid approach. A meta-aggregative synthesis (based on a qualitative approach) was performed.
Research sample	Not applicable
Sampling strategy	Not applicable
Data collection	Data was collected through querying respective databases and preprint servers. Data material was stored digital.
Timing	Data was collected in July 2023
Data exclusions	738 out of 796 records were excluded based on predefined inclusion and exclusion criteria.
Non-participation	Not applicable
Randomization	Not applicable

Reporting for specific materials, systems and methods

We require information from authors about some types of materials, experimental systems and methods used in many studies. Here, indicate whether each material, system or method listed is relevant to your study. If you are not sure if a list item applies to your research, read the appropriate section before selecting a response.

Materials & experimental systems

n/a	Involved in the study	
☒	☐	Antibodies
☒	☐	Eukaryotic cell lines
☒	☐	Palaeontology and archaeology
☒	☐	Animals and other organisms
☒	☐	Clinical data
☒	☐	Dual use research of concern
☒	☐	Plants

Methods

n/a	Involved in the study	
☒	☐	ChIP-seq
☒	☐	Flow cytometry
☒	☐	MRI-based neuroimaging

Plants

Seed stocks	Not applicable
Novel plant genotypes	Not applicable
Authentication	Not applicable
